# Cloning and Characterization of a Novel Vacuolar Na^+^/H^+^ Antiporter Gene (*Dgnhx1*) from Chrysanthemum

**DOI:** 10.1371/journal.pone.0083702

**Published:** 2013-12-20

**Authors:** Qing-Lin Liu, Ke-Dong Xu, Ming Zhong, Yuan-Zhi Pan, Bei-Bei Jiang, Guang-Li Liu, Yin Jia

**Affiliations:** 1 Department of Ornamental Horticulture, Sichuan Agricultural University, Chengdu, China; 2 Key Lab of Plant Genetics & Molecular Breeding of Department of Life Science, Zhoukou Normal University, Zhoukou, China; Purdue University, United States of America

## Abstract

Plant vacuolar Na^+^/H^+^ antiporter genes play significant roles in salt tolerance. However, the roles of the chrysanthemum vacuolar Na^+^/H^+^ antiporter genes in salt stress response remain obscure. In this study, we isolated and characterized a novel vacuolar Na^+^/H^+^ antiporter gene *DgNHX1* from chrysanthemum. The *DgNHX1* sequence contained 1920 bp with a complete open reading frame of 1533 bp encoding a putative protein of 510 amino acids with a predicted protein molecular weight of 56.3 kDa. *DgNHX1* was predicted containing nine transmembrane domains. Its expression in the chrysanthemum was up-regulated by salt stress, but not by abscisic acid (ABA). To assess roles of *DgNHX1* in plant salt stress responses, we performed gain-of-function experiment. The *DgNHX1*-overexpression tobacco plants showed significant salt tolerance than the wild type (WT). The transgenic lines exhibited more accumulation of Na^+^ and K^+^ under salt stress. These findings suggest that *DgNHX1* plays a positive regulatory role in salt stress response.

## Introduction

Salinity is one kind of environmental stress affecting plant growth and productivity worldwide. Plant can initiate an array of morphological, physiological, and biochemical adaptations to salt stress. In these adaptations, many salt stress-related genes are induced [1], [2]. Among numerous salt-induced genes, plant vacuolar Na^+^/H^+^ antiporter genes (*NHXs*) received much attention [3]–[7]. Plant vacuolar Na^+^/H^+^ antiporters play important roles in maintaining cellular ion homeostasis and mediating the transport of Na^+^ out of the cytosol and into the vacuole [8]. To date, a number of vacuolar Na^+^/H^+^ antiporter genes have been isolated and characterized from different plant species. Many studies have been reported to improve salt stress resistance of plants by over-expression of vacuolar Na^+^/H^+^ antiporter genes such as *AtNHX1*, *GhNHX1*, *OsNHX1*, and *EgNHX1* etc [3], [4], [6], [7]. All these results suggest that vacuolar Na^+^/H^+^ antiporter genes play significant roles in salt tolerance.

Chrysanthemum is one of the most famous ornamental species in the world and its production is severely affected by high salinity conditions in the cutting-chrysanthemum industry [9]–[11]. So far, no research related to the vacuolar Na^+^/H^+^ antiporter genes in chrysanthemum is available. In an objective of improving salt tolerance in chrysanthemum, we reported the cloning and characterization of a novel vacuolar Na^+^/H^+^ antiporter gene, *DgNHX1*, and showed that it was induced by salt stress. By stress assays, overexpression of *DgNHX1* in tobacco plants enhanced salt tolerance.

## Materials and Methods

### Plant materials and stress treatments

Chrysanthemum seedlings were grown in greenhouse. For salt and ABA treatments, seedlings at the six-leaf stage were incubated in 250 mM NaCl and 100 µM ABA solution respectively. Seedlings were sampled at 0, 3, 6, 12, 24, and 48 h after treatment, and immediately stored at −80°C for RNA extraction.

### Cloning of *DgNHX1* gene

Based on the transcriptomic data of chrysanthemum seedlings under normal conditions and salt treatment using 454 high throughout sequencing technique, numerous salt-induced transcripts were identified. Among these, one transcript, *DgNHX1*, was significantly induced by salt treatment. To obtain the full-length *DgNHX1*sequence, seedlings at the six-leaf stage were incubated in 250 mM NaCl for 24 h were harvested. Total RNA was extracted following the manufacturer's instructions (Mylab, Beijing). The full-length *DgNHX1* sequence was obtained by PCR and using the gene specific primers (Table S1).

### Gene expression analysis

For RT-PCR analysis, total RNA was extracted from plant samples following the manufacturer's instructions (Mylab, Beijing) and treated with RNase-free DNase I in accordance with manufacturer's instructions (Fermentas). Total RNA (1 ug) was reverse-transcribed in a 20 µl reaction mixture using the 1 µl superscript II enzyme (Invitrogen, USA). RT-PCR was performed for 34 and 29 amplification cycles for *DgNHX1* and *NtUbiquitin*, respectively.

Seedlings at the six-leaf stage were incubated in 250 mM NaCl or 100 µM ABA solution respectively, and the leaves were collected for measurement. Extraction of total RNAs and reverse transcription were performed as described above. Quantitative real-time PCR (qRT-PCR) analysis was performed as described previously [10], [11]. Relative expression levels were calculated by the 2^-△△CT^ method, where △△CT = (CT, Target-CT, actin gene) _the indicated time treatment_-(CT, Target-CT, actin gene)_0h treatment_ [12]. Three replicate biological experiments were conducted.

### Generation of *DgNHX1* transgenic tobacco

The *DgNHX1* cDNA was cloned into pBI121 (Clotech) under the control of the cauliflower mosaic virus (CaMV) 35S promoter via BamHI and SacI sites. The recombinant plasmid was introduced into tobacco through *Agrobacterium tumefaciens* strains GV3101-mediated leaf disc method [13].

### Analysis of salt tolerance in the *DgNHX1* transgenic tobacco plants

The T_2_ generation plants of lines OE-6, OE-9, and OE-13 were used in the subsequent experiments. For survival experiments, three-week-old seedlings of *DgNHX1* transgenic T_2_ lines and wild type tobacco plants were irrigated with 400 mM NaCl for 7 days. The survival rate of the seedlings as scored after a re-watering regularly as a recovery process for 6 days. Three replicate biological experiments were conducted.

### Measurement of Na^+^ and K^+^ contents

Three-week-old WT seedlings and T_2_ seedlings of *DgNHX1* transgenic tobacco plants lines OE-6, OE-9, and OE-13 were exposed to 400 mM NaCl for 96 h, and the leaves were harvested and then dried at 80°C for 24 h. The Na^+^ and K^+^ contents of the leaves were measured using atomic absorption spectrophotometry.

## Results

### Cloning and sequence analysis of *DgNHX1*

**Figure 1:**
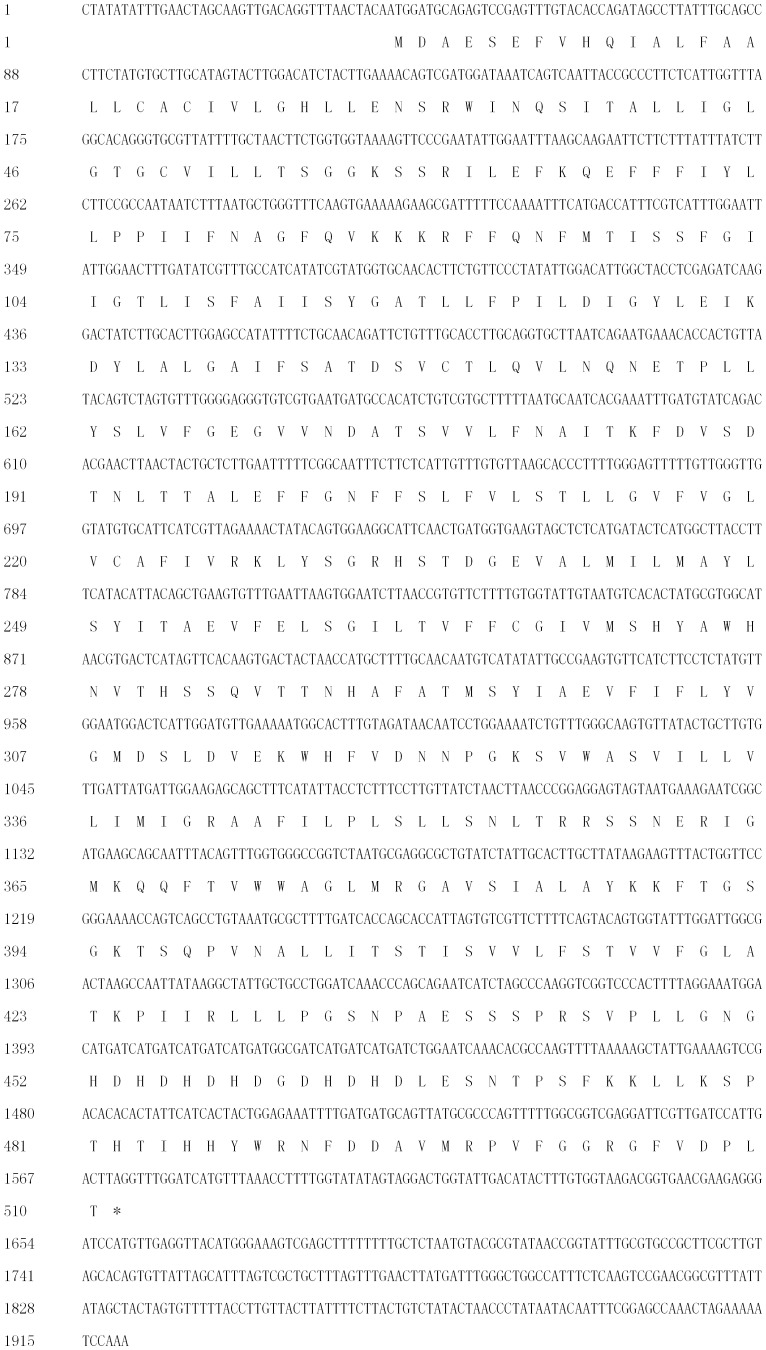
Nucleotide and deduced amino acid sequences of *DgNHX1*.

**Figure 2:**
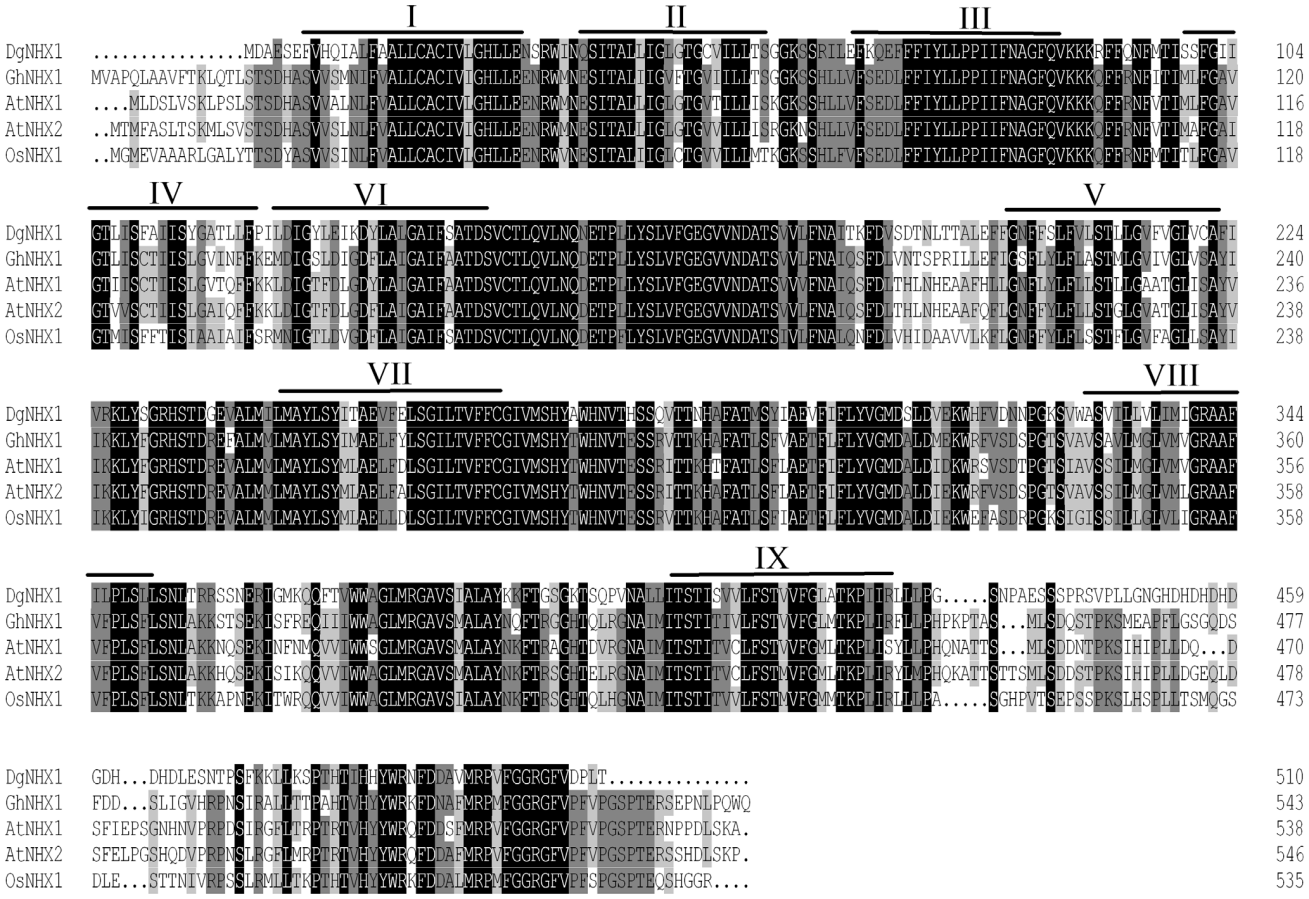
Amino acid sequences of DgNHX1 and other vacuolar Na^+^/H^+^ antiporter proteins from selected plant species. Alignments were performed using DNAMAN (version 6.0). Amino acid residues conserved in all four sequences were shaded in black, and those conserved in three sequences were shaded in light grey. The 9 transmembrane domains (Labeled as I–IX) of DgNHX1 were indicated by lines above the sequences.

**Figure 3:**
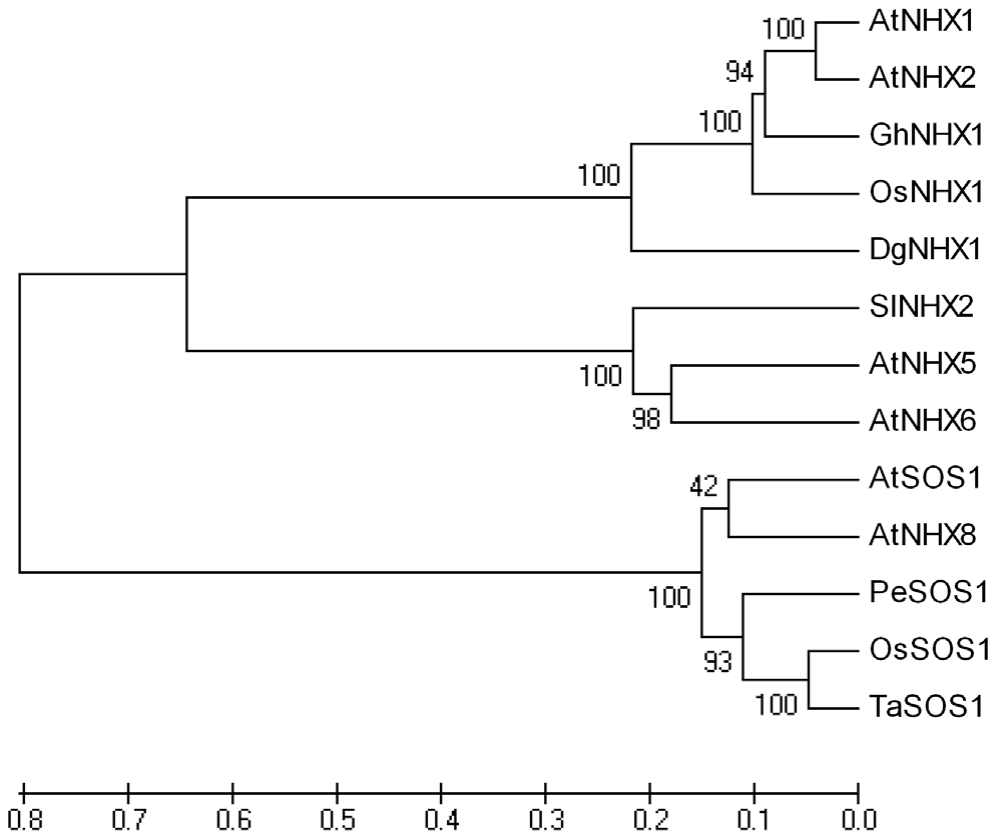
Phylogenetic tree of DgNHX1 and other vacuolar Na^+^/H^+^ antiporter proteins from other plant species. The tree was constructed by neighbor-joining method using MEGA software (ver 5). Branch numbers represent as percentage of bootstrap values in 1000 sampling replicates and scale indicates branch lengths. The accession numbers as follows: AtNHX1 (AAD16946), AtNHX2 (AAG51408), AtNHX5 (AAD25617), AtNHX6 (AAF68127), AtSOS1 (NP178307), AtNHX8 (NP172918) from *Arabidopsis thaliana*; GhNHX1 (AAM54141) from *Gossypium hirsutum*, OsNHX1 (BAA83337), OsSOS1 (AAW33875) from *Oryza sativa*; SlNHX2 (CAC83608), from *Solanum lycopersicum*; PeSOS1 (ABF60872) *Populus euphratica*, TaSOS1 (AAQ91618) from *Triticicum aestivum*.

The *DgNHX1* sequence contained 1920 bp with a complete open reading frame of 1533 bp encoding a putative protein of 510 amino acids with a predicted protein molecular weight of 56.3 kDa (Figure 1). Sequence comparison by DNAMAN (Version 6.0) revealed that DgNHX1 shared high homology with other vacuolar Na^+^/H^+^ antiporter proteins, such as OsNHX1 (60.56%), GhNHX1 (58.97%), AtNHX1 (58.44%), and AtNHX2 (58.67%), but it shared less homology with the plasma Na^+^/H^+^ antiporter AtSOS1 (10.55%). The hydropathy plot generated by the SOSUI program revealed that DgNHX1 contained nine transmembrane domains, and the third transmembrane domain contained a putative amiloride-binding domain LFFIYLLPPI (Figure 2). The phylogenetic analysis showed that DgNHX1 belongs to the vacuolar Na^+^/H^+^ antiporter proteins and is more closely related to OsNHX1, and GhNHX1 (Figure 3).

### Expression pattern of *DgNHX1* in different tissues and response to salt stress

**Figure 4:**
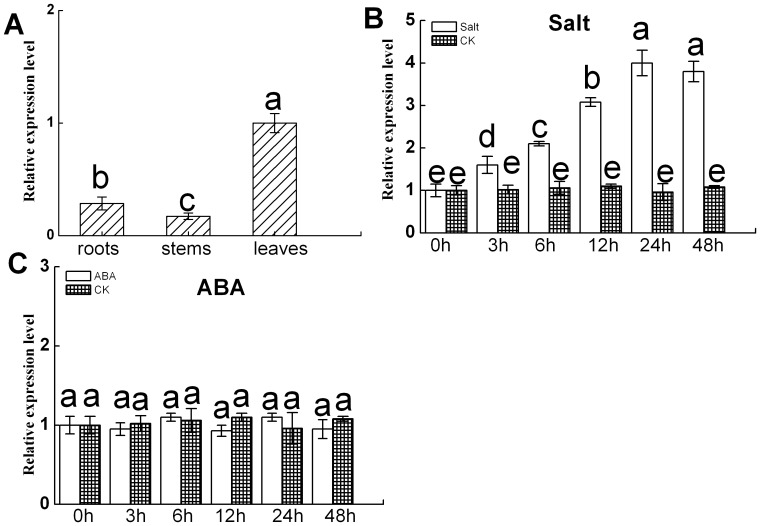
Quantitative real-time PCR analysis of expression patterns of *DgNHX1* in different organs and in response to salt and ABA treatments. The relative expression of *DgNHX1* in untreated leaves was used as CK. (A) Expression patterns of *DgNHX1* in roots, stems, and leaves. (B) Salt. (C) ABA. Data represent means and standard errors of three replicates. Different letters above columns indicate (P<0.05) differences between treatments.

The tissue specificity of *DgNHX1* transcript accumulation was examined by qRT-PCR method. As showed in Figure 4A, *DgNHX1* was expressed strongly in seedling leaves, weakly in seedling roots, and seedling stems under non-stressed conditions.

The expression levels of *DgNHX1* increased significantly under salt stress, but were not activated by ABA (Figure 4B and C). Under salt stress, *DgNHX1* transcript increased gradually up to 24 h after NaCl treatment, and thereafter decreased slightly (Figure 4B).

### Overexpression of *DgNHX1* enhanced tolerance to salt stress

**Figure 5:**
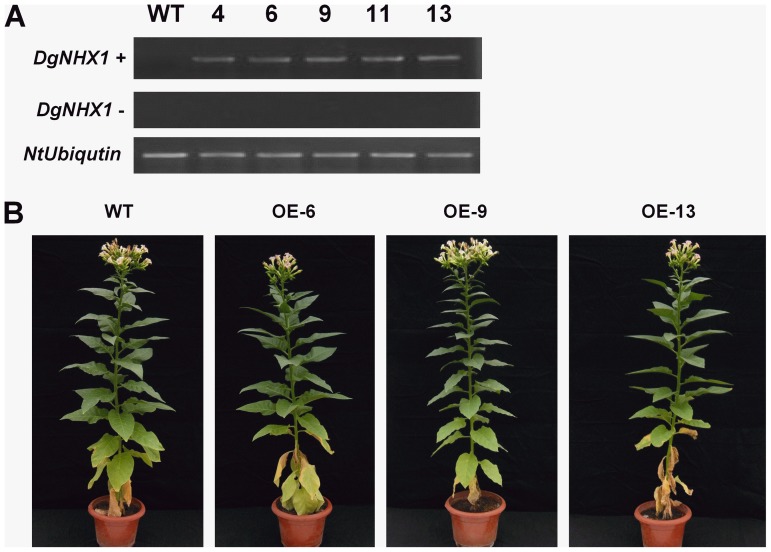
Phenotype of *DgNHX1*-overexpression transgenic tobacco plants (OE-6, OE-9, and OE-13). (A) The expression level of *DgNHX1* in WT and *DgNHX1*-OE transgenic T_2_ lines. Ethidium bromide staining of PCR products using *DgNHX1*-specific primers with (top) and without (middle) prior reverse transcription, and RT-PCR products with *NtUbiqutin*-specific primers (bottom). (B) Phenotype of *DgNHX1*-overexpression transgenic tobacco plants.

**Figure 6:**
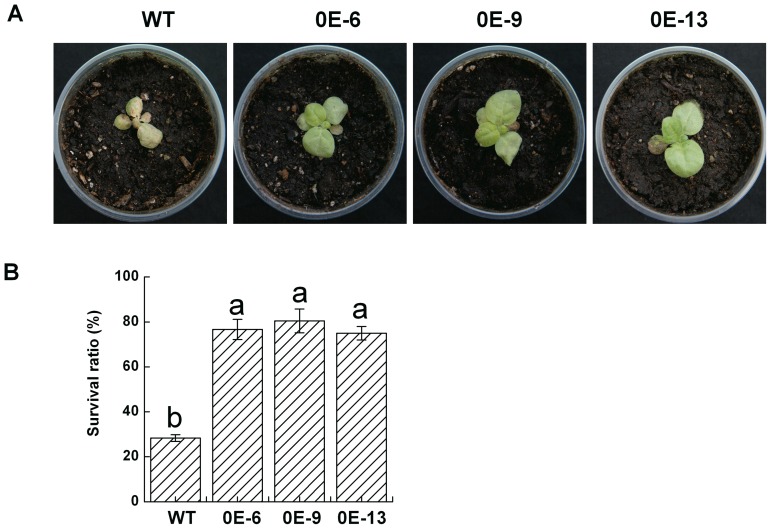
Analysis of salt tolerance in *DgNHX1*-overexpression transgenic tobacco plants (OE-6, OE-9, and OE-13). (A) The seedlings in WT and *DgNHX1*-OE transgenic T_2_ lines were irrigated with 400 mM NaCl for 7 days to assess physical symptoms. (B) Survival rates of seedling in wild type and *DgNHX1*-OE transgenic T_2_ lines after 6 days recovery. About 100 seedlings were used for each treatment. Different letters above columns indicate (P<0.05) significant differences according to Duncan's multiple range test between lines.

In order to analysis the function of *DgNHX1*, an overexpressing construct under the control of the CaMV 35S promoter, was transformed into tobacco plants. Among 22 lines of transformants, five independent transgenic lines (OE-4, OE-6, OE-9, OE-11, and OE-13) were confirmed by using RT-PCR analysis (Figure 5A). The T_2_ generation plants of lines OE-6, OE-9, and OE-13 were used in the subsequent experiments. Under normal growth conditions, no obvious differences were detected between the *DgNHX1*-overexpression and wild-type (WT) tobacco plants (Figure 5B). In the salt tolerance assay, three-week-old transgenic lines and WT were irrigated with 400 mM NaCl for 7 days. It was observed that the WT were more wilted than transgenic seedlings (OE-6, OE-9, and OE-13) (Figure 6A). After 6 days of recovery from salt stress, the three transgenic lines (OE-6, OE-9, and OE-13) showed significantly higher survival rates of 76%, 80%, and 75%, respectively, as compared to that of WT plants (28%) (Figure 6B).

### Analysis of accumulation of Na^+^ and K^+^ in *DgNHX1* transformed tobacco plants under salt stress

**Figure 7:**
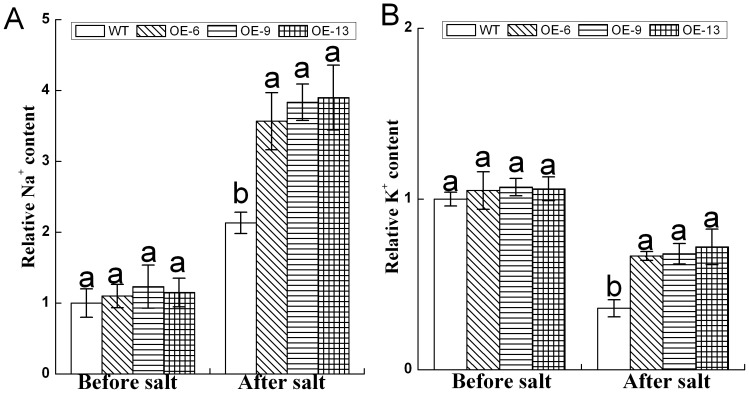
Na^+^ (A) and K^+^ (B) contents in leaves of WT and transgenic T_2_ lines (OE-6, OE-9, and OE-13) under normal conditions or after 4 days salt treatment, respectively. Data represent means and standard errors of three replicates. Different letters above columns indicate (P<0.05) significant differences according to Duncan's multiple range test between lines.

To investigate if overexpression of *DgNHX1* enhanced the Na^+^ and K^+^ accumulation in tobacco, the Na^+^ and K^+^ contents were measured. Under normal conditions, there was no significant difference in the Na^+^ and K^+^ contents between WT and three transgenic lines (Figure 7). Upon exposure to salt stress, there was a marked increase in Na^+^ content for both WT and the transgenic lines (Figure 7A). However, the accumulation of Na^+^ content was significantly higher in the three transgenic lines than WT in response to salt stress. In contrast, there was a marked decrease in K^+^ content for both WT and the transgenic lines, and the reduction of K^+^ content was significantly less in the three transgenic lines than WT in response to salt stress (Figure 7B).

## Discussion

Plant vacuolar Na^+^/H^+^ antiporter genes have been shown to play significant roles in salt tolerance [8]. A vacuolar Na^+^/H^+^ antiporter gene termed *DgNHX1* from chrysanthemum was cloned and characterized in this study. Sequence analysis showed that it contained nine transmembrane domains. The DgNHX1 was structurally similar to OsNHX1, which was isolated from *Oryza sativa* under salt stress [6], and GhNHX1, which was isolated from *Gossypium hirsutum* under salt stress [7]. These results indicate that *DgNHX1* is a novel member of the vacuolar Na^+^/H^+^ antiporter genes.

The phylogenetic analysis showed that DgNHX1 belongs to the vacuolar Na^+^/H^+^ antiporter proteins, which consists of several well-characterized vacuolar Na^+^/H^+^ antiporter genes, including *OsNHX1*, *GhNHX1*, and *AtNHX1*. In *Oryza sativa*, overexpression of *OsNHX1* also enhanced the tolerance to salt stress in transgenic lines [6]. Overexpression of another salt-induced vacuolar Na^+^/H^+^ antiporter protein gene, *GhNHX1*, has been reported to confer salt tolerance in tobacco plants [7]. In addition, overexpression *AtNHX1* improved salt tolerance in many plants [3], [14], [15]. Transcript levels of *DgNHX1* were increased by salt stress, and the *35S:DgNHX1* transgenic tobacco exhibited a markedly increased tolerance to salt. These results suggest that *DgNHX1* may be involved in salt tolerance.

The putative protein encoded by the *DgNHX1* gene shares 58.44% homology with AtNHX1, which may perform the similar function in salt tolerance improvement of the plants used. To overcome the issue of heterologous genetic transformation, *DgNHX1* might be better than *AtNHX1* for application in genetic engineering strategies aimed at improving salt stress tolerance in chrysanthemum.

*DgNHX1*-overexpression tobacco plants conferred salt tolerance and have no difference in phenotypes during all life cycles between the *DgNHX1*-overexpression and WT tobacco plants under normal conditions. It indicated that *DgNHX1* might be a potentially excellent genetic resource for the improvement of salt tolerance in chrysanthemum.

Under salt stress, *DgNHX1*-overexpression plants accumulate more Na^+^ and K^+^, as compared to that of WT plants. The increased accumulation of Na^+^ and K^+^ may be correlated with an increase activity of the vacuolar Na^+^/H^+^ antiporter, which plays important roles in the compartmentation of Na^+^ and K^+^ highly accumulated in the cytoplasm into the vacuoles [8], [16]. Similar observations were reported for other vacuolar Na^+^/H^+^ antiporter genes involved in salt stress, such as *AtNHX1* and *HcNHX1* [3], [17]. These results indicate that *DgNHX1* enhanced the accumulation of Na^+^ and K^+^ and resulted in the increased tolerance to salt stress.

ABA plays a crucial role in the adaptive response of plants to salt stress [18]. In this study, expression of *DgNHX1* was not responsive to ABA treatment. This result suggests that *DgNHX1* might be involved in an ABA-independent salt stress-responsive signal pathway.

In conclusion, this study cloned and characterized a vacuolar Na^+^/H^+^ antiporter gene, *DgNHX1*, which was induced by salt stress, was isolated from chrysanthemum. *DgNHX1*-overexpression tobacco plants enhanced the accumulation of Na^+^ and K^+^ and resulted in the increased tolerance to salt stress. Therefore, *DgNHX1* provides a promising tool for improving salt tolerance in chrysanthemum.

## Supporting Information

Table S1 **The primers used in the present study.**
(DOC)
