# Supplementary material for: Cloning and Characterization of a Novel Vacuolar Na+/H+ Antiporter Gene (Dgnhx1) from Chrysanthemum
Source: PLoS One. 2013 Dec 20;8(12):e83702. doi: 10.1371/journal.pone.0083702 (PMC3869812; doi:10.1371/journal.pone.0083702)
Supplement: Table S1 — The primers used in the present study. (DOC) [file pone.0083702.s001.doc]

**Supplementary Table S1.** The primers used in the present study

| **Primer Name Sequence(5′-3′) Description** | | |
| --- | --- | --- |
| A1  A2  A3  A4  A5  A6  B1  B2  B3  B4 | TGAACTAGCAAGTTGACAGGTTTAA  TTCACCGTCTTACCACAAAGTATG  AGCAGAATCATCTAGCCCAAG  GTGATGAATAGTGTGTGTCGGA  CCAGTGGTCGTACAACTGGCATT  CAGTCAGATCACGACCAGCAAGATC  CATTCAACTGATGGTGAAGTAGC  ATAATTGGCTTAGTCGCCAATC  TTAACACATGCAAGTCGGACG GAGACCTCAGTAGACAAAGCACATC | Full-length cDNA for *DgNHX1*  qRT-PCR for *DgNHX1*  qRT-PCR for chrysanthemum actin gene  RT-PCR for *DgNHX1*  RT-PCR for *NtUbiquitin* |
